# Supplementary material for: High-throughput viable circulating tumor cell isolation using tapered-slit membrane filter-based chipsets in the differential diagnosis of ovarian tumors
Source: PLoS One. 2024 Jun 4;19(6):e0304704. doi: 10.1371/journal.pone.0304704 (PMC11149860; doi:10.1371/journal.pone.0304704)
Supplement: S1 Table — (DOCX) [file pone.0304704.s001.docx]

| S1 Table. Characteristics for patients with benign tumor vs. stage I/II ovarian cancer | | | |
| --- | --- | --- | --- |
|  | Benign  (n = 81) | Stage I or II ovarian cancer  (n = 46) | P value |
| Age (years) |  |  | 0.030 |
| ≤ 48 | 53 (65.4) | 21 (45.7) |  |
| > 48 | 28 (34.6) | 25 (54.3) |  |
| CA-125 (U/mL) |  |  | 0.202 |
| ≤ 35 | 43 (53.1) | 19 (41.3) |  |
| >35 | 38 (46.9) | 27 (58.7) |  |
| CT or MRI |  |  | <0.001 |
| Benign | 50 (64.9) | 1 (2.3) |  |
| Borderline | 10 (13.0) | 4 (9.1) |  |
| Malignancy | 17 (22.1) | 39 (88.6) |  |
| Operation approach |  |  | <0.001 |
| Laparoscopic | 51 (63.0) | 10 (21.7) |  |
| Open | 30 (37.0) | 36 (78.3) |  |
| Tumor size (cm) |  |  | 0.002 |
| ≤ 11 | 61 (75.3) | 22 (47.8) |  |
| > 11 | 20 (24.7) | 24 (52.2) |  |
| Ascites |  |  | 0.620 |
| No | 79 (97.5) | 44 (95.7) |  |
| Yes | 2 (2.5) | 2 (4.3) |  |
| Values are presented as number (%).  CA, cancer antigen; CT, computerized tomography; MRI, magnetic resonance imaging | | | |
