# Supplementary material for: High-throughput viable circulating tumor cell isolation using tapered-slit membrane filter-based chipsets in the differential diagnosis of ovarian tumors
Source: PLoS One. 2024 Jun 4;19(6):e0304704. doi: 10.1371/journal.pone.0304704 (PMC11149860; doi:10.1371/journal.pone.0304704)
Supplement: S2 Table — (DOCX) [file pone.0304704.s002.docx]

| S2 Table. Clinical factors according to presence of preoperative CTCs | | | |
| --- | --- | --- | --- |
|  | CTC negative  N=85 | CTC positive  N=116 | P value |
| Age (years) |  |  | 0.059 |
| ≤ 48 | 51 (60.0) | 54 (46.6) |  |
| > 48 | 34 (40.0) | 62 (53.4) |  |
| CA-125 (U/mL) |  |  | 0.013 |
| ≤ 35 | 45 (52.9) | 41 (35.3) |  |
| >35 | 40 (47.1) | 75 (64.7) |  |
| CT or MRI |  |  | 0.004 |
| Benign to borderline | 44 (54.3) | 38 (33.6) |  |
| Malignancy | 37 (45.7) | 75 (66.4) |  |
| Tumor size (cm) |  |  | 0.593 |
| ≤ 11 | 53 (62.4) | 68 (58.6) |  |
| > 11 | 32 (37.6) | 48 (41.4) |  |
| Ascites |  |  | 0.034 |
| No | 79 (92.9) | 96 (82.8) |  |
| Yes | 6 (7.1) | 20 (17.2) |  |
| Values are presented as number (%).  CTC, circulating tumor cells; CA, cancer antigen; CT, computerized tomography; MRI, magnetic resonance imaging | | | |
