# Supplementary material for: High-throughput viable circulating tumor cell isolation using tapered-slit membrane filter-based chipsets in the differential diagnosis of ovarian tumors
Source: PLoS One. 2024 Jun 4;19(6):e0304704. doi: 10.1371/journal.pone.0304704 (PMC11149860; doi:10.1371/journal.pone.0304704)
Supplement: S3 Table — (DOCX) [file pone.0304704.s003.docx]

| S3 table. Univariate and multivariate logistic regression analyses of risk factors for presence of preoperative CTCs | | | | | | |
| --- | --- | --- | --- | --- | --- | --- |
|  | Univariate | | | Multivariate* | | |
|  | HR | 95% PI | P value | HR | 95% PI | P value |
| Age |  |  | 0.060 |  |  | 0.125 |
| ≤ 48 | 1 |  |  | 1 |  |  |
| > 48 | 1.722 | 0.977-3.036 |  | 1.613 | 0.876-2.971 |  |
| CA-125 |  |  | 0.013 |  |  | 0.149 |
| ≤ 35 | 1 |  |  | 1 |  |  |
| >35 | 2.058 | 1.162-3.643 |  | 1.578 | 0.849-2.935 |  |
| CT or MRI |  |  | 0.004 |  |  | 0.004 |
| Benign to borderline | 1 |  |  | 1 |  |  |
| Malignancy | 2.347 | 1.306-4.217 |  | 2.347 | 1.306-4.217 |  |
| Tumor size (cm) |  |  | 0.593 |  |  |  |
| ≤ 11 | 1 |  |  |  |  |  |
| > 11 | 1.169 | 0.659-2.075 |  |  |  |  |
| Ascites |  |  | 0.039 |  |  | 0.256 |
| No | 1 |  |  | 1 |  |  |
| Yes | 2.743 | 1.051-7.162 |  | 1.804 | 0.651-4.998 |  |
| * Backward, conditional method  CTC, circulating tumor cells; CA, cancer antigen; CT, computerized tomography; MRI, magnetic resonance imaging | | | | | | |
